# Molecular bases of morphologically diffused tumors across multiple cancer types

**DOI:** 10.1093/nsr/nwac177

**Published:** 2022-08-26

**Authors:** Dingyun Liu, Feiyang Xing, Yueying Wang, Jun Xiao, Zheng An, Ying Xu

**Affiliations:** College of Computer Science and Technology, Jilin University, China; Center for Cancer Systems Biology, China-Japan Union Hospital of Jilin University, China; Center for Cancer Systems Biology, China-Japan Union Hospital of Jilin University, China; School of Life Sciences, Jilin University, China; College of Computer Science and Technology, Jilin University, China; College of Computer Science and Technology, Jilin University, China; Center for Cancer Systems Biology, China-Japan Union Hospital of Jilin University, China; Center for Cancer Systems Biology, China-Japan Union Hospital of Jilin University, China; Computational Systems Biology Lab, Department of Biochemistry and Molecular Biology and Institute of Bioinformatics, University of Georgia, USA; Center for Cancer Systems Biology, China-Japan Union Hospital of Jilin University, China; Computational Systems Biology Lab, Department of Biochemistry and Molecular Biology and Institute of Bioinformatics, University of Georgia, USA

**Keywords:** cancer morphology, diffuse cancers, cancer malignancy, sialic acid

## Abstract

Gastric cancer has two distinct subtypes: the diffuse (DGC) and the intestinal (IGC) subtypes. Morphologically, the former each consists of numerous scattered tiny tumors while the latter each has one or a few solid biomasses. The former tends to be more aggressive and takes place in younger patients than the latter. While these have long been documented, little is known about the underlying causes. Our hypothesis is that the level of sialic acid (SA) accumulation on the cancer cell surfaces is a key reason for the observed differences. Our transcriptomic data-based analyses provide evidence that (i) DGCs tend to deploy more SAs on cancer cell surfaces than IGCs; (ii) this gives rise to considerably stronger cell–cell electrostatic repulsion in DGCs due to the negative charge that each SA carries; and (iii) such repulsion drives stronger cell protrusion and metastasis. Similar observations as well as our transcriptomic data-based predictions hold for multiple other cancer types, namely breast, lung, prostate plus liver and thyroid cancers, each known to have diffuse-like vs. non-diffused subtypes as well as more aggressive behaviors like DGCs vs. IGCs. Hence, we speculate that the discovery presented here applies not only to gastric cancer but multiple and even potentially all cancer types having diffuse-like and non-diffused subtypes.

Diffuse gastric cancer (DGC) and intestinal gastric cancer (IGC) are the two main gastric cancer subtypes [1]. Morphologically, DGC consists of numerous disjoint tiny tumors while IGC forms one or a few solid tumors (Supplementary Supplementary Fig. S1) [1,2]. In-depth studies have discovered that DGCs generally have an ill-defined or missing glandular structure and reduced cell–cell adhesion, and may tend to consist of a large fraction of signet cells [3] compared to IGCs. They also tend to be more aggressive and happen to younger patients compared to IGCs [1]. A number of studies have been published regarding the genomic and transcriptomic differences between DGC and IGC [4,5]. However, no studies have established how these molecular-level differences are functionally linked to the distinct morphologies and aggressiveness between DGCs and IGCs.

We have discovered based on transcriptomic data analyses that DGCs generally accumulate considerably more sialic acids (SAs) on the cancer cell surfaces compared to IGCs, which will result in stronger cell–cell electrostatic repulsion since SAs each carry a negative charge and hence on average larger cell–cell distances similar to that among red blood cells. Statistical analyses reveal that there is a strong association between the elevated expression levels of the relevant genes and the reduced survival time. These lead to our main hypothesis: *the level of SA accumulation on the cancer cell surfaces is a key factor that dictates the morphology and the aggressiveness of DGCs vs. IGCs*.

Literature search has revealed that multiple other cancer types also each consist of subtypes like diffuse vs. non-diffused tumors of gastric cancer, namely scattered tiny tumors with ill-defined glandular structures, summarized in Supplementary Supplementary Table S1. Our further analyses provided strong evidence that this hypothesis applies to these cancer types as well.

## MORE SAs ON CELL SURFACES IMPLY STRONGER CELL–CELL REPULSION AND METASTATIC POTENTIAL

It has long been observed since the 1960s that overproduction of SAs, which are the capping molecules of cell-surface glycans, is associated with cancer metastasis in general [6]. Each SA carries a negative charge and hence its over-deployment on the cell surfaces will lead to stronger electrostatic repulsion among neighboring cells, similar to red blood cells (RBCs) [7], which are known to deploy significantly more SAs on their surface than other cell types, preventing them from aggregation [8]. Hence, we have examined the expression levels of genes relevant to the SA accumulation on the cell surfaces, namely the sialyltransferase (ST) genes for deploying SAs and sialidase genes for degrading SAs.

**Figure 1.**
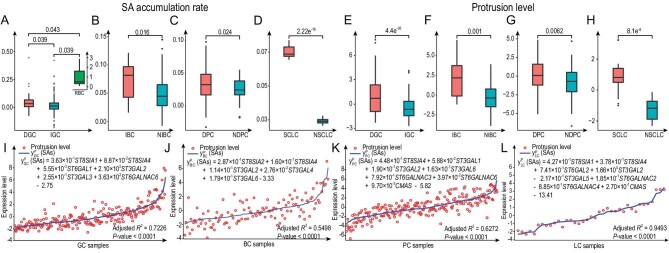
Key statistical results. (A) The predicted SA accumulation rates in DGC, IGC and RBC samples. (B) The predicted SA accumulation rates in IBC and NIBC samples. (C) The predicted SA accumulation rates in DPC and NDPC samples. (D) The predicted SA accumulation rates in SCLC and NSCLC samples. (E) The estimated protrusion levels in DGCs and IGCs. (F) The estimated protrusion levels in IBCs and NIBCs. (G) The estimated protrusion levels in DPCs and NDPCs. (H) The estimated protrusion levels in SCLCs and NSCLCs. (I) Model fitting for protrusion levels on GCs. The red circles represent the protrusion level of GC reflected by the expression of the protrusion-related genes; the blue curve represents the predicted protrusion level *y* ^P^_GC_(SAs); and ST gene names denote their respective expression levels. GC samples were listed in the ascending order of their *y* ^P^_GC_(SAs) values from left to right along the *x*-axis. For the corresponding density distribution curves of protrusion levels and *y* ^P^_GC_(SAs) values, *x*-axis denotes expression level and *y*-axis denotes sample proportion. The green curves *P*_diffuse_ (*x*) denote the probability of the sample with protrusion level *x* [or *y* ^P^_GC_(SAs) value *x*] being the diffuse-like sample, calculated from the corresponding the density distribution. Similar is defined and performed for BC, PC and LC samples in (J), (K) and (L), respectively. Detailed methods are given in Supplementary material.

We note that DGC samples (Supplementary Table S2) generally have higher levels of ST gene expressionsand lower sialidase gene expressions, suggesting that DGCs have more SAs accumulated on their cell surfaces than IGCs (Supplementary Table S3). This is supported by a quantitative analysis using the Michaelis–Menten kinetic model [7] (Supplementary material), which suggests that the SA accumulation rate and hence the cell–cell repulsion in DGCs are significantly higher than those of IGCs (Fig. 1A and Supplementary material).

Knowing that DGCs have higher death rates compared to IGCs [1], we have then analysed the levels of cell protrusion: a key migration-related activity [7] (Supplementary material), since metastasis is the predominant reason for cancer death, accounting for >93% of cancer-related death. Our analyses show that the protrusion level is considerably higher in DGCs than in IGCs (Fig. 1E). A linear regression analysis confirms that the increased protrusion activity can be statistically well explained by the expressions of the ST genes in DGCs (Fig. 1I and Supplementary material). This is further supported by the survival data associated with integrin genes in DGCs and IGCs (Supplementary Table S4, Supplementary Fig. S2 and Supplementary material).

## YOUNGER DGC PATIENTS ARE DUE TO THE SPECIFIC GROWTH FACTOR USED

Our previous study suggests that more malignant cancers tend to happen in younger patients [9]. Specifically, a cancer requires two types of factors to take place, one being the cancer risk factor in an organ, which goes up with age, and the other the availability level of circulatory growth factors specifically needed by a cancer type, which decreases with age.

Using the program developed in [9], we have predicted the growth factors specifically needed by DGCs and IGCs, respectively (Supplementary material), with *PDGFC * being the main growth factor needed by DGCs and *EREG* and *NRG2* the growth factors needed by IGCs (Supplementary Table S3 and Supplementary material), which is supported by published studies (Supplementary Table S5) and by an accurate regression analysis (Supplementary Fig. S3 and Supplementary material). Similar is done for IGCs (Supplementary Fig. S3 and Supplementary material). Furthermore, the average level of growth factor *PDGFC* drops more sharply from the population with age <60 years to that with age >60 years than those of *EREG* and *NRG2*, hence providing a natural explanation for the observation (Supplementary material).

## COMPARATIVE ANALYSES ON OTHER CANCER TYPES

Literature search has revealed that multiple other cancer types also each have tumor subtypes similar to DGCs in terms of their morphology and aggressiveness, as summarized in Supplementary Table S1. For each such cancer type, we call their DGC-like tumors diffuse-like tumors. Specifically, breast cancer (BC), prostate cancer (PC) and lung cancer (LC) each have a relatively large number of diffuse-like tumor samples with transcriptomic data in the public domain (Supplementary Table S2). In addition, other cancer types recorded in Supplementary Table S1 also have very limited diffuse-like tumor samples but we do not include them in the following analyses as their numbers of samples are too small.

### SA deployment

The same analyses were conducted on BC, PC and LC. Inflammatory breast cancer (IBC), PC with Gleason score of ≥8 (DPC) and small-cell lung carcinoma (SCLC) are considered to be diffuse-like subtypes of BC, PC and LC, respectively, while non-IBC (NIBC), PCs with Gleason score of ≤6 (NDPC) and non-SCLC (NSCLC) are the corresponding non-diffused subtypes (Supplementary Table S1). Our analyses have revealed that for each of the three cancer types, its diffuse-like tumors, on average, have higher levels of ST gene expressions (Supplementary Table S3). And Michaelis–Menten kinetics-based calculations show that the SA accumulation rates and cell–cell repulsion in the three diffuse-like tumors are consistently higher than those of non-diffused tumors (Fig. 1B–D and Supplementary material). Furthermore, the level of the predicted cell–cell repulsion is consistent with the survival rate of each diffuse-like subtype (Supplementary Table S1).

### Increased metastatic potential

Analyses were then conducted to estimate the level of cell protrusion in the diffuse-like vs. non-diffused BC, PC and LC tumors, respectively. Comparable results are achieved here compared to those for DGCs vs. IGCs (Fig. 1F–H). In addition, the increased protrusion activities in diffuse-like tumors can be statistically well explained by the expressions of the SA synthesis gene and ST genes for each of the three cancer types (Fig. 1J–L and Supplementary material). Based on these, we predict that the elevated SA deployment plays important roles in the increased migration activities and metastatic potentials of the diffuse-like subtypes of all three cancer types, which is further supported by published studies (Supplementary Table S5).

## SUMMARY

Our data analyses and computational modeling provide support for the hypothesis that the distinct levels of SA accumulation on cancer cell surfaces are the key reason for the different morphologies and the aggressiveness between diffuse-like and non-diffused tumors of the same cancer types, hence providing new insights for an important and fundamental cancer biology question for the first time. The key novelty of our study lies in the fact that higher levels of the SA accumulation, estimated based on transcriptomic data, coupled with a physics-based argument provide a natural explanation for the possible causes of the distinct morphology as well as aggressiveness between diffuse-like vs. non-diffused tumors, plus the generality of this discovery. Clearly, this is a computation-based discovery. Further validation is needed by physically measuring the levels of cell–cell repulsion in diffuse-like vs. non-diffused tumors and establishing the detailed relationship between the repulsion levels and the tumor sizes.

## DATA AVAILABILITY

The data used in this work are openly and freely available in TCGA (https://portal.gdc.cancer.gov/), GEO (https://www.ncbi.nlm.nih.gov/geo/), SEER (https://seer.cancer.gov/), GTEx (https://gtexportal.org/home/), HUMAN PROTEIN ATLAS (https://www.proteinatlas.org/), Bionumbers (https://bionumbers.hms.harvard.edu/), and UALCAN (http://ualcan.path.uab.edu/).

## Supplementary Material

nwac177_Supplemental_Files
