# Supplementary material for: Molecular bases of morphologically diffused tumors across multiple cancer types
Source: Natl Sci Rev. 2022 Aug 26;9(11):nwac177. doi: 10.1093/nsr/nwac177 (PMC9744092; doi:10.1093/nsr/nwac177)
Supplement: nwac177_Supplemental_Files [file nwac177_supplemental_files.zip › Supplementary_Material-revised.docx]

**Molecular Bases of Morphologically Diffused Tumors across Multiple Cancer Types**

Dingyun Liu^1,2^, Feiyang Xing^1,3^, Yueying Wang^2^, Jun Xiao^1,2^, Zheng An^1,4^, Ying Xu^1,4,*^

^1^Center for Cancer Systems Biology, China-Japan Union Hospital of Jilin University, China

^2^College of Computer Science and Technology, Jilin University, China

^3^School of Life Sciences, Jilin University, China

^4^Computational Systems Biology Lab, Department of Biochemistry and Molecular Biology and Institute of Bioinformatics, the University of Georgia, USA

* Correspondence: xyn@uga.edu.

**SUPPLEMENTARY MATERIAL**

**DATA**

Gene expression data:

258 samples of RNA-seq data of gastric cancer (GC) are retrieved from the TCGA database. Of these, 63 are diffuse gastric cancer (DGC), 163 are intestinal gastric cancer (IGC), and 32 are matching control tissues, *i.e.*, solid normal tissues, denoted by SNT. Their sample ID is summarized in Table S6A. Read-count values are used in our differential expression analyses and TPM values are used for other computational analyses, namely Pearson correlation calculation, expression level of gene sets, and regression analyses.

RNA-seq data of diffuse-like and non-diffused samples of breast cancer (BC) and prostate cancer (PC) are also downloaded from TCGA database, both Read-count and TPM values are used. As summarized in Tables S2, S6B and S6C. Microarray data of diffuse-like and non-diffused lung cancer (LC) samples are downloaded from the GEO dataset (GSE40275), also shown in Tables S2 and S6D. The diffuse natures of BC are not recorded in the original TCGA clinical data. Therefore, we utilized the identification results in [1] to classify the BC samples into diffuse-like and non-diffused groups.

RNA-seq data of seven red blood cell (RBC) samples are downloaded from the GEO dataset (GSE108378, and GSE63703) (Table S6E).

GC data need for age analysis:

GC risk data are collected from the SEER database [2]. Growth-factor receptor and cell-cycle genes are collected from our previous study [3]. Gene expression data used to estimate the age-dependent synthesis rate of each target growth factor are from GTEx, reprocessed by UCSC Xena [4, 5], as described in [3].

The data used in this work are openly and freely available in TCGA [6], GEO [7], SEER [2], GTEx [4], HUMAN PROTEIN ATLAS [8] Bionumbers [9], and UALCAN [10].

**METHODS**

**Basic gene-expression analysis tools**

Differentially expressed genes (DEGs) are calculated by read-count values of the RNA-seq data when comparing expressions among or between the DGC, IGC and SNT samples. “TCGAanalyze_DEA” in the R package “TCGAbiolinks” is used [11]. Genes with *P*-values < 0.05, log2 CPM > 0 and |log2 FC| ≥ 0.5 are regarded as DEGs. The DEGs between DGC-IGC, DGC-SNT, and IGC-SNT are summarized in Tables S3A - S3C. DEGs between diffuse-like and non-diffused breast cancer (i.e. inflammatory breast cancer (IBC), and non-IBC (NIBC)), and diffuse-like and non-diffused prostate cancer (DPC and NDPC), are also calculated by the same method, recorded in Tables S3D - S3E.

DEGs between diffuse-like and non-diffused lung cancer (i.e. small-cell lung carcinoma (SCLC), and non-SCLC (NSCLC)) are calculated using the corresponding microarray expression data, via the functions “lmFit”, “eBayes”, and “topTable” in the R package “limma” [12]. Results are shown in Table S3F.

Function “corr.test” in the R package “psych” is used for calculation of the Pearson correlation coefficient, *PCC*. Expression vector pairs with *PCC* > 0.3 and *P*-value < 0.0001 are regarded as statistically correlated unless stated otherwise.

**Estimating the expression level of a gene set**

For each gene set, we identify a maximum subset of its genes, called the signature genes, whose expressions correlates with at least one other gene of the subset. Given an expression matrix ***G*** = c[***g***_1_; ***g***_2_;...***g***_k_], where ***g****_i_* is the row vector of the expression values (TPM for GC, BC, and PC samples, and microarray value for LC samples) of the *i*^th^ signature gene over diffuse-like or non-diffused cancer samples, *i* = 1, 2, ..., *k*. The combined Z-score *Z*_s_ [13] of ***G*** is defined as:

$Z_{s}=\frac{\sum_{i=1}^{k} Z_{i}}{\sqrt{k}}$ (1)

where *Z*_1_, *Z*_2_, ..., *Z_k_* are the z-scores of ***g***_1_, ***g***_2_, ..., ***g***_k_, respectively. This method was used to obtain the level of migration-related cell protrusion activity (Figure 1), protrusion genes are collected from [14].

**Estimation of sialic acid (SA) accumulation rate for cancer cells and RBCs**

*a. Expression data normalization*

We have collected the expression of 18,504 protein-coding genes to form the expression matrix for GC, BC, PC, LC, and RBC samples. Then, function “ComBat-seq” in the R package “sva” is applied to the read-count expression matrix to remove the batch effect for GC, BC, PC and RBC samples. We then convert the read-count values to protein-TPM (pTPM) values, to normalize the total expression of protein-coding genes for each sample. For LC samples, we directly use the microarray expression data of the protein-coding genes to perform the following calculations.

*b. Estimation of SA accumulation rate*

Knowing that the SA accumulation rate depends on the rates of SA synthesis, degradation, and transfer to cell-surface, we have used the normalized pTPM or microarray expressions of the following genes to make the estimation: *CMAS* for SA synthesis, sialyltransferase (ST) genes for poly-SA transfer or transfer to gangliosides (*ST8SIA1*, *ST8SIA2*, *ST8SIA3*, *ST8SIA4*, *ST8SIA5*, *ST3GAL1*, *ST3GAL2*, *ST3GAL5*, *ST6GALNAC3*, *ST6GALNAC5*, and *ST6GALNAC6*), *NEU1* and *CTSA* for SA degradation, where *CTSA* is needed to form a complex with *NEU1* for SA degradation [14]. Hence, the rate of SA placement onto cell surface is estimated via the following function:

$R_{\text{SAp}}=\sqrt{CMAS\times\sum_{i=1}^{11} \text{ST}_{i}}$ (2)

and the rate of SA degradation is estimated as follows:

$R_{\text{SAd}}=\sqrt{NEU1\times CTSA}$ (3)

where each gene name represents its expression level and is SA transfer rate, ST*_i_* refers to the *i*^th^ ST gene.

This model is established based on the following assumption: for a given enzyme gene *X* with expression level *E*(*X*), the maximum reaction rate of the enzyme encoded by *X* is proportional to K_cat_ × *E*(*X*), with K_cat_ being the reaction rate constant by enzyme *X* in the Michaelis-Menten formulation. This is essentially equivalent to the assumption that (i) the expression level of a gene is proportional to its protein concentration; and (ii) the reactant concentration is higher than the reaction constant K_M_, a common assumption used when modeling human metabolism [14]. Knowing that the that *CMAS*, ST genes and *NEU1* have comparable reaction rate constant K_cat_ values [14-16], we can use linear models to estimate the rates of SA placement and degradation. In addition, the geometric average of the two functions is used based on the following consideration: this form has been widely used to estimate the number of complexes formed by two molecular species [14, 17]. Knowing that the two functions (2) and (3) are both monotonically increasing of their vairables, the detailed form should not affect our main estimation results on SA placement and degradation. After the above calculations, we use following function to estimate the SA accumulation rate for cancer cells and RBCs:

$R_{\text{SAa}}=\frac{R_{\text{SAp}}-R_{\text{SAd}}}{\text{S}_{\text{c}}}$ (4)

where S_c_ is the average surface area of a cell type, collected from Bionumbers. For RBCs, S_c_ is about 140 μm^2^; for GCs, BCs, PCs, and NSCLCs (non-diffused type of LC) it is about 1600 μm^2^, approximated using that of HeLa cells, for SCLCs, the published study has reported that the average nuclear diameter of them is about 63% of that of NSCLCs [18]. Therefore, the estimated average surface area for SCLC cells is about 40% of that of NSCLC cells, in this work we set this parameter to 700 μm^2^.

The *R*_SAa_ values for DGC, IGC, and RBC samples are shown in Figure 1A. The medians of *R*_SAa_ for DGC, IGC, and RBC samples are 0.0262, 0.0089, and 0.5057, respectively. The *R*_SAa_ values for IBC and NIBC, DPC and NDPC, SCLC and NSCLC samples are shown in Figures 1B - 1D. The corresponding medians for IBC and NIBC samples are 0.0820 and 0.0450, respectively. Those for DPC and NDPC are 0.0326 and 0.0246, respectively. And Those for SCLC and NSCLC are 0.0688 and 0.0292, respectively. Therefore, the level of cell-cell repulsion between DGC cells is more than four times of that between IGC cells with the same distance, knowing that each SA carries a negative charge, giving rise to electrostatic repulsion force between two cells is proportional to the product of the total surface charge of each cell. Similarly, level of cell-cell repulsion in IBC is more than three times of that in NIBC of the same distance; that in DPC is 70% more than that in NDPC; and that in SCLC is more than five times of that in NSCLC.

**Regression analysis of migration-related cell protrusion activity**

A regression analysis was conducted over cancer samples of the levels of migratory behavior-protrusion against the expression level of SA synthesis gene (*CMAS*), and ST genes, through function “regsubsets” in the R package “leap”. Bayesian Information criterion, Mallows’ Cp and adjusted *R*^2^ values are used to assess the quality of the regression model. Genes chosen by the final regression model are deemed as the key promoters to cancer cells’ protrusion. We finally obtain the following analytic function for DGCs and IGCs:

*y*^P^_GC_(SAs) = 3.6294 × 10^-1^ *ST8SIA1* + 8.8697 ×10^-2^ *ST8SIA4*

+ 5.5447 × 10^-3^ *ST6GAL1* + 2.0986 ×10^-2^ *ST3GAL2* (5)

+ 2.5538 × 10^-1^ *ST3GAL3* + 3.6322 ×10^-2^ *ST6GALNAC6* - 2.7518

where *y*^P^_GC_(SAs) denotes the approximate level of protrusion based on the expressions of the ST genes, and ST gene names denote their respective TPM-based expressions. The approximation accuracy of this function is adjusted *R*^2^ = 0.7226 with *P*-value < 0.0001 (Figure 1I).

In the same way, we obtained the analytic function for IBCs and NIBCs’ protrusion level:

*y*^P^_BC_(SAs) = 2.8731 × 10^-1^ *ST8SIA2* + 1.6018 ×10^-1^ *ST8SIA4*

+ 1.1356 × 10^-1^ *ST3GAL2* + 2.7564 ×10^-2^ *ST3GAL4* (6)

+ 1.7853 × 10^-1^ *ST3GAL6* – 3.3342

The approximation accuracy of this function is adjusted *R*^2^ = 0.5498 with *P*-value < 0.0001 (Figure 1J).

Similarly, we obtained the analytic functions for DPCs and NDPCs:

*y*^P^_PC_(SAs) = 4.4780 × 10^-1^ *ST8SIA4* + 5.8811 × 10^-2^ *ST3GAL1*

+ 1.9040 × 10^-1^ *ST3GAL2* + 1.6334 × 10^-1^ *ST3GAL6* (7)

+ 7.9223 × 10^-1^ *ST6GALNAC3* + 3.9676 × 10^-2^ *ST6GALNAC6*

+ 9.6956 × 10^-3^ *CMAS* – 5.8181

The approximation accuracy of the function *y*^P^_PC_(SAs) is 0.6272 with qualified *P*-value < 0.0001 (Figure 1K).

For SCLC and NSCLC, we obtained the following analytic functions:

*y*^P^_LC_(SAs) = 4.2719 × 10^-1^ *ST8SIA1* + 3.7781 × 10^-1^ *ST8SIA4*

+ 7.4091 × 10^-1^ *ST6GAL2* + 1.6582 × 10^0^ *ST3GAL2* (8)

- 2.1702 × 10^-1^ *ST3GAL5* + 1.8517 × 10^-1^ *ST6GALNAC2*

- 8.8467 × 10^-1^ *ST6GALNAC4* + 2.6967 × 10^-1^ *CMAS* – 13.4104

Where ST gene names denote their respective microarray-based expressions. The approximation accuracy of the function *y*^P^_LC_(SAs) is 0.9493 with qualified *P*-value < 0.0001 (Figure 1L).

**Calculation of probability of a sample being diffuse-like**

Let *P*_diffuse_(*x*) be the probability of the sample with protrusion level *x* (or *y*^P^_GC_(SAs), *y*^P^_BC_(SAs), *y*^P^_PC_(SAs), and *y*^P^_LC_(SAs) value *x*) being a diffuse-like sample, it is calculated as follows:

*P*_diffuse_(*x*) = *D*_diffuse_(*x*) / (*D*_diffuse_(*x*) + *D*_non-diffused_(*x*)), (9)

where *D*_diffuse_ (*x*) is the density value of *x* in the red curve (diffuse-like) of the density distribution. Similarly, *D*_non-diffuse_(*x*) is the density value of *x* in the cyan curve (non-diffuse), as shown in Figure 1I - 1L.

**Analysis of integrin gene expressions**

We have examined the expressions of integrin genes in both DGCs and IGCs (Tables S4A - S4D). We have noted: four *ITGA* genes, namely *ITGA1*, *5*, *9* and *V* are consistently upregulated in DGC vs. IGC in at least three of the four stages. *ITGB1* has higher expression levels in DGC vs. IGC across all four stages; and *ITGB4* has lower expression levels in DGC vs. IGC across all four stages. These genes all have strong implications to survival. Specifically, upregulated *ITGA1*, *5*, *9*, *V* and *ITGB1* all imply reduced survival rates for gastric cancer patients while upregulated *ITGB4* implies an improved survival rate of gastric cancer patients (available from [10], *P*-value < 0.1, and Figure S2). Furthermore, integrin pairs *ITGA2*-*ITGB1*, *ITGA6*-*ITGB1* and *ITGA6*-*ITGB4* are known metastasis suppressors in various cancers [19-21], and all are downregulated in DGC vs. IGCs in at least three of the four stages.

In addition, all these integrin genes show strong correlations with at least three of the five ST genes which upregulated in DGCs vs. IGCs, except for the downregulated *ITGB4*. Particularly, *ITGA1*, 5, and 9 show high correlations with *ST3GAL3* and *ST6GALNAC5* (*PCC* > 0.6 with *P*-value < 0.0001, Tables S4E - S4F).

**Identification of growth factor for a cancer type**

The basic idea is to (i) first identify growth factor receptors that are upregulated in cancer tissues (vs. SNT) of a specific type, whose expressions strongly correlate with the cell-cycle genes in the same cancer tissues; and (ii) each growth factor is available in blood circulation. Using this method, we have predicted growth factors needed by DGC and IGC tissues. Then, the growth factors with average TPM expressions < 10 at any age are discarded [3], and thus we identified the growth factors for DGC and IGC, with names and the corresponding receptors given in Table S3B and S3C.

**Prediction of main contributors of DGC and IGC occurrences**

A regression analysis is performed, using “regsubsets” in the R package “leap”, of the age-dependent occurrence rate of each gastric cancer type against (i) the age-dependent risk of occurrence of the cancer and (ii) the age-dependent blood concentrations of the growth factors, as described in [3], to predict growth factors specifically needed a cancer type. The age-dependent occurrence rates of DGCs and IGCs are not available in the SEER database, therefore it is approximated by the proportions of TCGA samples at age 31-40, 41-50, ..., 81-90 (Figure S3A). The corresponding age-dependent cancer risk is approximated by the risk of GC given in the SEER database. Age-dependent circulatory level curves of the target ligands are collected from [3]. Bayesian Information criterion, Mallows’ Cp and adjusted *R*^2^ values are used to assess the final regression model and the growth factors selected in the final regression model are regarded as the main contributors to the occurrence rate of each cancer type.

Growth factors in DGC: To facilitate the regression analysis, we have utilized the following analytic function:

*y*^D^(*x*) = - 4.5100 × 10^-4^ *x*^2^ + 5.4762 × 10^-2^ *x* -1.3652 (10)

to approximate the age-specific DGC occurrence distribution collected as a series of discrete data, where *x* denotes age, ranging from 35 to 85. The approximation accuracy of this analytic function has adjusted *R*^2^ = 0.9402 with *P*-value = 0.006798 vs. the actual age information of the DGC samples (Figure S3B).

The number of stem-cell divisions in stomach from birth to the current age, retrieved from the SEER reports, were used to estimate the age-dependent gastric cancer risk, which is approximated using the following analytic function:

*f*_1_(*x*) = 2.2643 × 10^−6^ *x*^3^ − 1.0147 × 10^−4^ *x*^2^ + 3.3456 × 10^−4^ *x* + 2.1440 × 10^-2^ (11)

where *x* denotes age. The approximation accuracy of this function has adjusted *R*^2^ =0.9989 with *P*-value < 0.0001 (Figure S3B).

Using the growth factors recorded in Table S3B, the regression model ultimately selected one protein, *PDFGC* as the main growth factor in DGC. The following function is used to approximate its (relative) age-dependent level in blood, which achieves the approximation accuracy at adjusted *R*^2^ = 0.9965 with *P*-value < 0.0001 against the age-specific data collected from the GTEx database (Figure S3B):

*f*_2_^D^(*x*) = -1.8899 × 10^−2^ *x*^2^ + 1.7958 *x* - 4.2469 (12)

and the following is the final regression model *y*^D^*’*(*x*) for DGC occurrence rate *y*^D^(*x*):

*y*^D^*’*(*x*) = 1.6619 × *f*_1_(*x*) + 8.8553 × 10^-3^ *f*_1_(*x*) *f*_2_^D^(*x*) + 5.0420 × 10^-2^ *f*_2_^D^(*x*) -1.8188 (13)

where *f*_1_(*x*)*f*_2_^D^(*x*) represents an interaction term between the two functions, whose regression accuracy has adjusted *R*^2^ = 0.9997 with *P*-value < 0.0001 (Figure S3B).

Growth factors in IGC: Similar to that of DGC, we have used the age-dependent distribution data in Figure S3A to estimate the relative age-dependent occurrence rate of IGC, which can be approximated using the following analytic function:

*y*^I^(*x*) = - 1.8742 × 10^-10^ *x*^5^ + 1.8571 × 10^-2^ *x* + 0.6755 (14)

with approximation accuracy adjusted *R*^2^ = 0.8847 and *P*-value = 0.01819 (Figure S3C). For age-dependent risk of IGC, the same function *f*_1_(*x*) (see Equation (11)) is used here.

A similar regression analysis of the cancer occurrence rates against the cancer risk and growth-factor availabilities leads to the selection of two growth factors, *EREG* and *NRG2*, whose age-dependent availabilities can be approximated by the following two analytic functions:

*f*_2_^I^(*x*) = -9.1474 × 10^−7^ *x*^5^ + 2.4352 × 10^−4^ *x*^4^ *-* 2.5006 × 10^−2^ *x*^3^

+ 1.2355 *x*^2^ - 29.4641 *x* + 2.9683 × 10^2^ (15)

*f*_3_^I^(*x*) = -6.9893 × 10^−7^ *x*^5^ + 1.9351 × 10^−4^ *x*^4^ *-* 2.0737 × 10^−2^ *x*^3^

+ 1.0679 *x*^2^ - 26.2237 *x* + 2.5558 × 10^2^ (16)

with approximation accuracy adjusted *R*^2^ = 0.9641 and *P*-value < 0.0001 for *EREG*; and adjusted *R*^2^ = 0.8354 and *P*-value < 0.0001 for *NRG2* (Figure S3C); and the following is the final regression model *y*^I^*’*(*x*) for IGC occurrence rate *y*^I^(*x*):

*y*^I^*’*(*x*) = 3.0546 × *f*_1_(*x*) - 5.9455 × 10^-1^ *f*_1_(*x*) *f*_2_^I^(*x*) + 9.0034 × 10^-1^ *f*_1_(*x*) *f*_3_^I^(*x*)

+ 1.4292 × 10^-2^ *f*_2_^I^(*x*) + 5.8635 × 10^-2^ *f*_3_^I^(*x*) - 9.9194 × 10^-1^ (17)

which achieves a regression accuracy at adjusted *R*^2^ = 0.9993 and *P*-value < 0.0001 (Figure S3C). This suggests that *EREG* and *NRG2* are the main growth factors used to drive cell proliferation in IGC.

We have calculated the average levels for the predicted growth factors for DGC and IGC in two age groups: 40 - 59 and 60 - 80, denoted by *C*_40-59_ and *C*_60-80_, respectively. Define *D* = (*C*_40-59_ - *C*_60-80_) / *C*_40-59_ × 100% as a quantity for the level of drop for a given growth factor in DGC or IGC. We note that PDGFC drops by *D* = 25.32% while the levels of the two growth factors for IGC drops lower than 10%, as shown in the following table:

**Table SA.** Average concentrations and level of drop for growth factors *PDGFC*, *EREG*, and *NRG2*

|  | *C*_40-59_ | *C*_60-80_ | *D* (%) |
| --- | --- | --- | --- |
| *PDGFC* | 37.7087 | 28.1593 | 25.32 |
| *EREG* | 22.7194 | 20.8435 | 8.26 |
| *NRG2* | 12.7244 | 11.5445 | 9.27 |

**REFERENCES**

1. Zare, A, Postovit, LM, Githaka, JM. Robust inflammatory breast cancer gene signature using nonparametric random forest analysis. *Breast Cancer Res*. 2021; **23**(1): 92.

2. Park, HS, Lloyd, S, Decker, RH*, et al.* Overview of the Surveillance, Epidemiology, and End Results database: evolution, data variables, and quality assurance. *Curr Probl Cancer*. 2012; **36**(4): 183-90.

3. Qiu, S, An, Z, Tan, R*, et al.* Understanding the unimodal distributions of cancer occurrence rates: it takes two factors for a cancer to occur. *Brief Bioinform*. 2020.

4. Consortium, GT. The Genotype-Tissue Expression (GTEx) project. *Nat Genet*. 2013; **45**(6): 580-5.

5. Goldman, MJ, Craft, B, Hastie, M*, et al.* Visualizing and interpreting cancer genomics data via the Xena platform. *Nat Biotechnol*. 2020; **38**(6): 675-8.

6. Tomczak, K, Czerwinska, P, Wiznerowicz, M. The Cancer Genome Atlas (TCGA): an immeasurable source of knowledge. *Contemp Oncol (Pozn)*. 2015; **19**(1A): A68-77.

7. Barrett, T, Wilhite, SE, Ledoux, P*, et al.* NCBI GEO: archive for functional genomics data sets--update. *Nucleic Acids Res*. 2013; **41**(Database issue): D991-5.

8. Uhlen, M, Fagerberg, L, Hallstrom, BM*, et al.* Proteomics. Tissue-based map of the human proteome. *Science*. 2015; **347**(6220): 1260419.

9. Milo, R, Jorgensen, P, Moran, U*, et al.* BioNumbers--the database of key numbers in molecular and cell biology. *Nucleic Acids Res*. 2010; **38**(Database issue): D750-3.

10. Chandrashekar, DS, Bashel, B, Balasubramanya, SAH*, et al.* UALCAN: A Portal for Facilitating Tumor Subgroup Gene Expression and Survival Analyses. *Neoplasia*. 2017; **19**(8): 649-58.

11. Colaprico, A, Silva, TC, Olsen, C*, et al.* TCGAbiolinks: an R/Bioconductor package for integrative analysis of TCGA data. *Nucleic Acids Res*. 2016; **44**(8): e71.

12. Smyth, GK. Limma: linear models for microarray data. *Bioinformatics and computational biology solutions using R and Bioconductor*: Springer; 2005. 397-420.

13. Lee, E, Chuang, HY, Kim, JW*, et al.* Inferring pathway activity toward precise disease classification. *PLoS Comput Biol*. 2008; **4**(11): e1000217.

14. Sun, H, Zhou, Y, Jiang, H*, et al.* Elucidation of Functional Roles of Sialic Acids in Cancer Migration. *Front Oncol*. 2020; **10**: 401.

15. Ortiz-Soto, ME, Seibel, J. Expression of Functional Human Sialyltransferases ST3Gal1 and ST6Gal1 in Escherichia coli. *PLoS One*. 2016; **11**(5): e0155410.

16. Thon, V, Lau, K, Yu, H*, et al.* PmST2: a novel Pasteurella multocida glycolipid alpha2-3-sialyltransferase. *Glycobiology*. 2011; **21**(9): 1206-16.

17. Vandesompele, J, De Preter, K, Pattyn, F*, et al.* Accurate normalization of real-time quantitative RT-PCR data by geometric averaging of multiple internal control genes. *Genome Biol*. 2002; **3**(7): RESEARCH0034.

18. Lee, TK, Silverman, JF, Horner, RD*, et al.* Overlap of nuclear diameters in lung cancer cells. *Anal Quant Cytol Histol*. 1990; **12**(4): 275-8.

19. Ishii, Y, Ochiai, A, Yamada, T*, et al.* Integrin alpha6beta4 as a suppressor and a predictive marker for peritoneal dissemination in human gastric cancer. *Gastroenterology*. 2000; **118**(3): 497-506.

20. Shimizu, H, Seiki, T, Asada, M*, et al.* Alpha6beta1 integrin induces proteasome-mediated cleavage of erbB2 in breast cancer cells. *Oncogene*. 2003; **22**(6): 831-9.

21. Ramirez, NE, Zhang, Z, Madamanchi, A*, et al.* The alpha(2)beta(1) integrin is a metastasis suppressor in mouse models and human cancer. *J Clin Invest*. 2011; **121**(1): 226-37.

**SUPPLEMENTARY FIGURES AND CAPTIONS**

**Figure S1.** Visuals of IGC and DGC (DOI:10.5772/intechopen.69825.) **A**. Gastrectomy of IGC. **B**. Morphology of a IGC tissue in **A**, showing adenocarcinoma. Haematoxylin-eosin, original magnification (OM) 100×. **C.** Gastrectomy of DGC. **D**. Morphology of a DGC tissue in **C**, showing signet ring cells. Immunoperoxidase, cytokeratin AE1/AE3, OM 400×. (Figure S1-ExampleofDGCandIGCtissues(supplementary data).jpg)

**Figure S2.** Kaplan-Meier survival curves of integrin genes and ST genes in TCGA, downloaded from UALCAN server. **A**. *ITGA1*. **B**. *ITGA5*. **C**. *ITGA9*. **D**. *ITGAV*. **E**. *ITGB1*. **F**. *ITGB4*. **G**. *ST8SIA4* in STAD. **H**. *ST6GALNAC6* in STAD. **I**. *ST3GAL3* in STAD. **J.** *ST3GAL3* in KIRC. **K**. *ST3GAL3* in ACC. **L**. *ST3GAL3* in CHOL. **M**. *ST8SIA1* in KIRP. **N**. *ST3GAL2* in BLCA. **O.** *ST8SIA4* in COAD. **P.** *ST8SIA1* in KICH. **Q.** *ST6GALNAC5* in MESO. **R**. *ST8SIA4* in UVM. **S**. *ST6GALNAC4* in LUSC. **T.** *ST3GAL5* in LUSC. **U**. *ST3GAL2* in THCA. **V.** *ST3GAL5* in LIHC. **W**. *ST6GALNAC4* in LIHC. **X**. *ST3GAL2* in LIHC. **Y**. *ST3GAL1* in BRCA. **Z**. *ST6GAL1* in BRCA. (Figure S2-UALCANsurvival(supplementary data).pdf)

**Figure S3.** Model fitting for DGC and IGC age-dependent occurrence rates. **A**. Age distribution of DGC and IGC patients/samples in TCGA. **B**. Model fitting for age-dependent DGC occurrence rates. *X*-axis is for age. The orange curve represents the predicted DGC incidence (*y*^D^’(*x*)). The black curve is for the known occurrence rate of DGC (*y*^D^(*x*)). The red curve is for the age-dependent cancer risk in stomach *f*_1_(*x*). The blue curve denotes the circulatory availability level of growth factor *PDGFC* (*f*_2_^D^(*x*)). The green curve is for the calculated age-dependent level of *PDGFC* & risk interactions (*f*_1_(*x*)*f*_2_^D^(*x*)). **C.** Model fitting for IGC occurrence rates. *X-*axis is for age. The orange curve denotes the predicted IGC incidence (*y*^I^’(*x*)). The black curve is for the known occurrence rate of IGC (*y*^I^(*x*)). The red curve represents the cancer risk in stomach *f*_1_(*x*). The blue and purple curves are for the availability levels of *EREG* (*f*_2_^I^(*x*)) and *NRG2* (*f*_3_^I^(*x*)), respectively. The green and cyan curves represent the calculated level of *EREG* & risk interactions (*f*_1_(*x*)*f*_2_^I^(*x*)) and *NRG2*-risk interactions (*f*_1_(*x*)*f*_3_^I^(*x*)), respectively. (Figure S3-Modelfitting-DGCIGCOccurrenceRates(supplementary data).pdf)

**SUPPLEMENTARY TABLES AND CAPTIONS**

**Table S1.** Diffuse-like tumors of 12 cancer types. (Table S1-Summary-DiffuseLiketumors(supplementary data).docx)

**Table S2.** Samples of diffuse-like and non-diffused subtypes of cancers used in this work. (Table S2- SampleSubtypes(supplementary data)..xlsx)

**Table S3.** Differentially expressed genes. **A**. Between DGC and IGC samples. **B**. Between DGC and SNT samples. **C**. Between IGC and SNT samples. **D**. Between IBC and NIBC samples. **E**. Between DPC and NDPC samples. **F**. Between SCLC and NSCLC samples. (Table S3-DEGs-GC-BC-PC-LC(supplementary data).xlsx)

**Table S4.** Integrin gene expressions in sample across four stages of gastric cancer and *PCC* between the five STs and integrin genes. **A.** stage I. **B**. stage II. **C**. stage III. **D**. stage IV. **E.** *PCC* values. **F**. Corresponding *P*-values. (Table S4-IntegrinData(supplementary data).xlsx)

**Table S5.** Supporting evidence for predicted growth factors and STs. **A**. Supporting evidence for *PDGFC* (key growth factor of DGCs). **B.** Supporting evidence for *EREG* and *NRG2* (key growth factor of IGCs). **C**. Supporting evidence for *ST8SIA2* and *ST8SIA4* (poly-SA). **D**. Supporting evidence for *ST3GAL3* and *ST3GAL4* (Sialyl-Lewis X). **E**. Supporting evidence for *ST8SIA1*, *ST6GALNAC5*, and *ST6GALNAC6* (disialyl-gangliosides) (Table S5- LiteratureEvidence(supplementary data).xlsx)

**Table S6.** Sample IDs used in this work. **A**. TCGA gastric cancer samples. **B**. TCGA breast cancer samples. **C**. TCGA prostate cancer samples. **D**. GEO lung cancer samples. **E**. GEO RBC samples. (Table S6-SampleID(supplementary data).xlsx)
