# Supplementary material for: Molecular bases of morphologically diffused tumors across multiple cancer types
Source: Natl Sci Rev. 2022 Aug 26;9(11):nwac177. doi: 10.1093/nsr/nwac177 (PMC9744092; doi:10.1093/nsr/nwac177)
Supplement: nwac177_Supplemental_Files [file nwac177_supplemental_files.zip › Table_S1-Summary-DiffuseLiketumors(supplementary_data).docx]

**Table S1**. Diffuse-like tumors of 12 cancer types.

| Cancer types | Information of diffuse-like tumors |
| --- | --- |
| Gastric  cancer | **Diffuse gastric cancer (DGC)** consists of numerous disjoint tiny tumors infiltrating the tissue stroma. Tumor cells are often poorly differentiated and lack cell-to-cell interactions. Glandular structures are rarely seen in DGC. Some of DGC tissues contain more than 50% signet-ring cells, referred to as signet-ring cell carcinoma. DGCs account for 32% of all gastric cancers [1], and their average survival rate is lower than other GCs [2]. |
| Breast  cancer | There are two types of diffuse-like breast cancers, accounting for < 3% of all breast cancers with significantly lower survival rates than other breast cancers [3] [4].  **Inflammatory breast cancer (IBC)** is the most aggressive entity of breast cancer. Compared to other breast tumors, IBC appears with acute inflammatory changes, and generally poorly differentiated with diffuse brawny infiltration, showing no underlying mass [3, 5, 6]. |
|  | **Signet-ring cell carcinoma (SRCC)** is defined according to the WHO’s classification as a poorly cohesive carcinoma in which more than 50% of tumor cells are with prominent cytoplasmic mucin and a crescent-shaped nucleus eccentrically placed. They are poorly differentiated and present as single cells or in loose clusters [4, 7]. |
| Prostate cancer | There are two types of diffuse-like prostate cancers, accounting for < 6% of all prostate cancers with significantly lower survival rates than other prostate cancers [4] [8].  Prostate cancers with **Gleason grade** (GG, from 1 - 5) ≥ 4 are defined as poorly differentiated tumors growing in a diffuse pattern. Tissues with GG 4 show poorly formed glandular lumina, while those in GG 5 are without glandular differentiation. Therefore, a tumor with **Gleason score** (GS, sum of the primary and secondary GG, range from 2-10) ≥ 8 with GGs ≥ 4 is considered as a diffuse-like tumor [8, 9]. |
|  | **SRCC** cancer tissues are derived from prostate epithelial cells [4, 7]. |
| Lung  cancer | There are two types of diffuse-like lung cancers, accounting for < 15% of all lung cancers with significantly lower survival rates than other lung cancers [4] [10, 11].  **Small-cell lung carcinoma (SCLC)** is defined as tumors with cells having a relatively small size, a round-to-fusiform shape, scant cytoplasm, and absent or inconspicuous nucleoli. The tumors are mostly poorly differentiated**,** fall short of glandular differentiation, and grow in diffuse sheets. [12, 13] |
|  | **SRCC** cancer tissues are derived from lung epithelial cells [4, 7]. |
| Liver  cancer | **Diffuse hepatocellular carcinoma (DHCC)** also known as cirrhotomimetic HCC or cirrhosis-like HCC, is a subtype of liver cancer. Different from massive HCC, DHCC shows cirrhosis-like diffuse growth. Cancer tissues exhibit pseudoglandular and trabecular patterns and may lack well-demarcated boundary [14-16]. DHCC often blend into the background of the cirrhotic liver. It accounts for < 20% of all HCC [14, 15] with significantly lower survival rates than other HCCs [17]. |
| Thyroid  cancer | **Diffuse sclerosing variant papillary thyroid carcinoma (DSVPTC)** is characterized by histologic features of numerous psammoma bodies, extensive lymphocytic infiltration, squamous metaplasia, diffuse fibrosis, calcification, and absence of string colloids together. DSVPTC, with highly diffuse appearance, can involve the thyroid gland extensively without forming a dominant mass [18, 19]**.** This subtype accounts for < 7% of all thyroid cancers [18] with significantly lower survival rates than classic papillary thyroid carcinoma [20]. |
| Colon  Cancer | **SRCC** cancer tissues are derived from colon epithelial cells [4, 7]. It accounts for < 1% of all colon cancers with significantly lower survival rates than non-SRCC adenocarcinomas [4]. |
| Pancreatic  Cancer | **SRCC** cancer tissues are derived from pancreatic epithelial cells [4, 7]. It accounts for < 1% of all pancreatic cancers with significantly lower survival rates than non-SRCC pancreatic cancers [4]. |
| Esophageal  Cancer | **SRCC** cancer tissues are derived from esophagus epithelial cells [4, 7]. It accounts for 2.6% of all esophageal cancers with significantly lower median survival than non-SRCC adenocarcinomas [4]. |
| Bladder  Cancer | **SRCC** cancer tissues are derived from bladder epithelial cells [4, 7]. It accounts for < 1% of all bladder cancers with significantly lower survival rates than non-SRCC adenocarcinomas [4]. |
| Gallbladder  Cancer | **SRCC** cancer tissues are derived from gallbladder epithelial cells [4, 7]. It accounts for 1.3% of all gallbladder cancers with significantly lower survival rates than non-SRCC adenocarcinomas [4]. |
| Ovarian  Cancer | **SRCC** cancer tissues are derived from ovary epithelial cells [4, 7]. It accounts for < 1% of all ovarian cancers with significantly lower survival rates than non-SRCC adenocarcinomas [4]. |

1. Hu, B, El Hajj, N, Sittler, S*, et al.* Gastric cancer: Classification, histology and application of molecular pathology. *J Gastrointest Oncol*. 2012; **3**(3): 251-61.

2. Chen, YC, Fang, WL, Wang, RF*, et al.* Clinicopathological Variation of Lauren Classification in Gastric Cancer. *Pathol Oncol Res*. 2016; **22**(1): 197-202.

3. Cristofanilli, M, Valero, V, Buzdar, AU*, et al.* Inflammatory breast cancer (IBC) and patterns of recurrence: understanding the biology of a unique disease. *Cancer*. 2007; **110**(7): 1436-44.

4. Benesch, MGK, Mathieson, A. Epidemiology of Signet Ring Cell Adenocarcinomas. *Cancers (Basel)*. 2020; **12**(6).

5. Jaiyesimi, IA, Buzdar, AU, Hortobagyi, G. Inflammatory breast cancer: a review. *J Clin Oncol*. 1992; **10**(6): 1014-24.

6. van Uden, DJ, van Laarhoven, HW, Westenberg, AH*, et al.* Inflammatory breast cancer: an overview. *Crit Rev Oncol Hematol*. 2015; **93**(2): 116-26.

7. Nagtegaal, ID, Odze, RD, Klimstra, D*, et al.* The 2019 WHO classification of tumours of the digestive system. *Histopathology*. 2020; **76**(2): 182-8.

8. Pierorazio, PM, Walsh, PC, Partin, AW*, et al.* Prognostic Gleason grade grouping: data based on the modified Gleason scoring system. *BJU Int*. 2013; **111**(5): 753-60.

9. Ahmed, HU, Arya, M, Freeman, A*, et al.* Do low-grade and low-volume prostate cancers bear the hallmarks of malignancy? *Lancet Oncol*. 2012; **13**(11): e509-17.

10. Gazdar, AF, Bunn, PA, Minna, JD. Small-cell lung cancer: what we know, what we need to know and the path forward. *Nat Rev Cancer*. 2017; **17**(12): 725-37.

11. Heineman, DJ, Daniels, JM, Schreurs, WH. Clinical staging of NSCLC: current evidence and implications for adjuvant chemotherapy. *Ther Adv Med Oncol*. 2017; **9**(9): 599-609.

12. Travis, WD. Update on small cell carcinoma and its differentiation from squamous cell carcinoma and other non-small cell carcinomas. *Mod Pathol*. 2012; **25 Suppl 1**: S18-30.

13. Raso, MG, Bota-Rabassedas, N, Wistuba, II. Pathology and Classification of SCLC. *Cancers (Basel)*. 2021; **13**(4).

14. Clayton, EF, Malik, S, Bonnel, A*, et al.* Liver transplantation and cirrhotomimetic hepatocellular carcinoma: classification and outcomes. *Liver Transpl*. 2014; **20**(7): 765-74.

15. Reynolds, AR, Furlan, A, Fetzer, DT*, et al.* Infiltrative hepatocellular carcinoma: what radiologists need to know. *Radiographics*. 2015; **35**(2): 371-86.

16. Jakate, S, Yabes, A, Giusto, D*, et al.* Diffuse cirrhosis-like hepatocellular carcinoma: a clinically and radiographically undetected variant mimicking cirrhosis. *Am J Surg Pathol*. 2010; **34**(7): 935-41.

17. Benvegnu, L, Noventa, F, Bernardinello, E*, et al.* Evidence for an association between the aetiology of cirrhosis and pattern of hepatocellular carcinoma development. *Gut*. 2001; **48**(1): 110-5.

18. Chereau, N, Giudicelli, X, Pattou, F*, et al.* Diffuse Sclerosing Variant of Papillary Thyroid Carcinoma Is Associated With Aggressive Histopathological Features and a Poor Outcome: Results of a Large Multicentric Study. *J Clin Endocrinol Metab*. 2016; **101**(12): 4603-10.

19. Vickery, AL, Jr., Carcangiu, ML, Johannessen, JV*, et al.* Papillary carcinoma. *Semin Diagn Pathol*. 1985; **2**(2): 90-100.

20. Al-Qahtani, KH, Al Asiri, M, Tunio, M*, et al.* Diffuse sclerosing variant papillary thyroid carcinoma: clinicopathological and treatment outcome analysis of 44 cases. *Kuwait Medical Journal*. 2015; **47**(3): 225-30.
