# Supplementary figures and images for: Molecular bases of morphologically diffused tumors across multiple cancer types
Source: Natl Sci Rev. 2022 Aug 26;9(11):nwac177. doi: 10.1093/nsr/nwac177 (PMC9744092; doi:10.1093/nsr/nwac177)

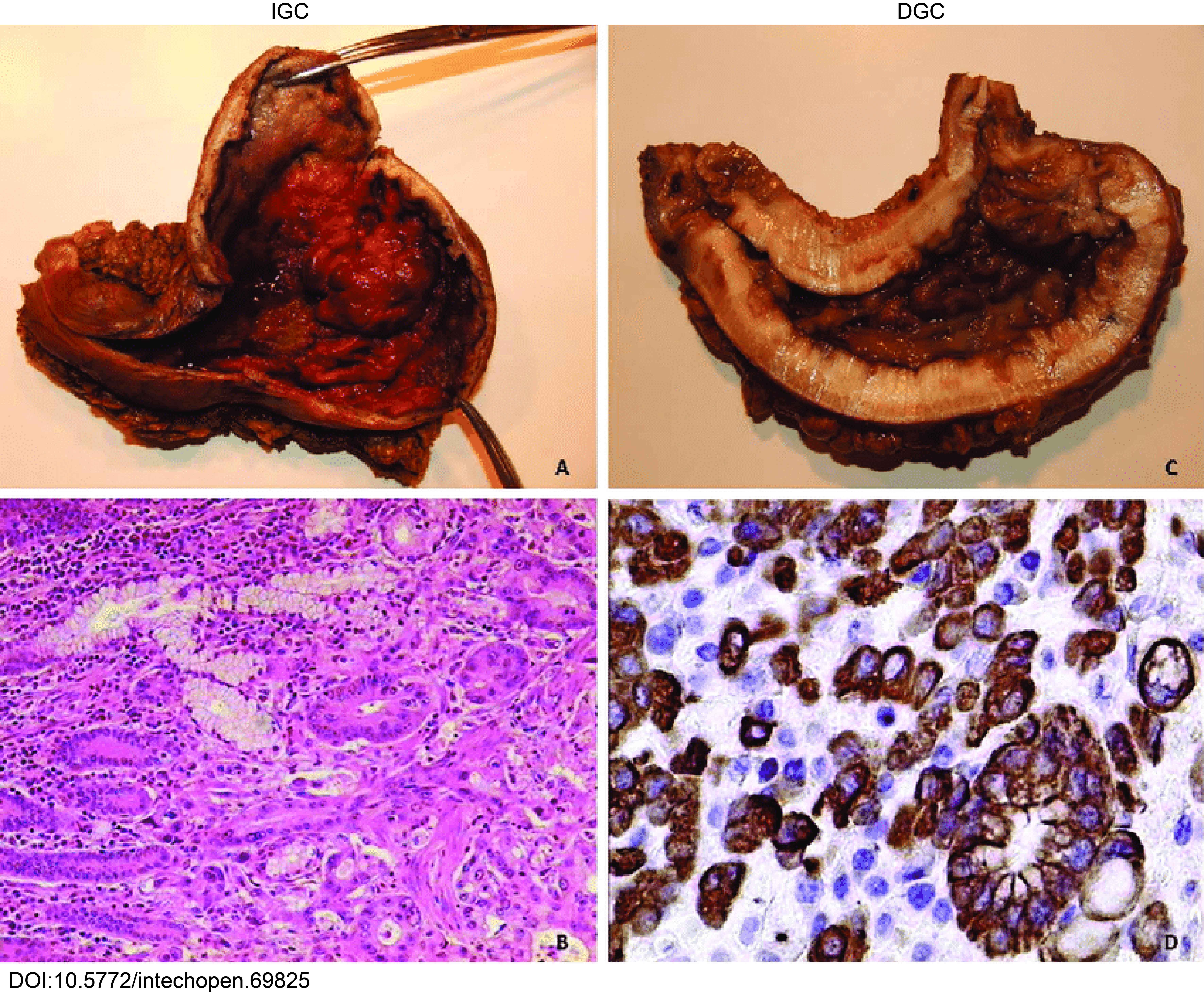

Supplement: nwac177_Supplemental_Files [file nwac177_supplemental_files.zip › Figure_S1-ExampleofDGCandIGCtissues(supplementary_data).jpg]

# Integrins

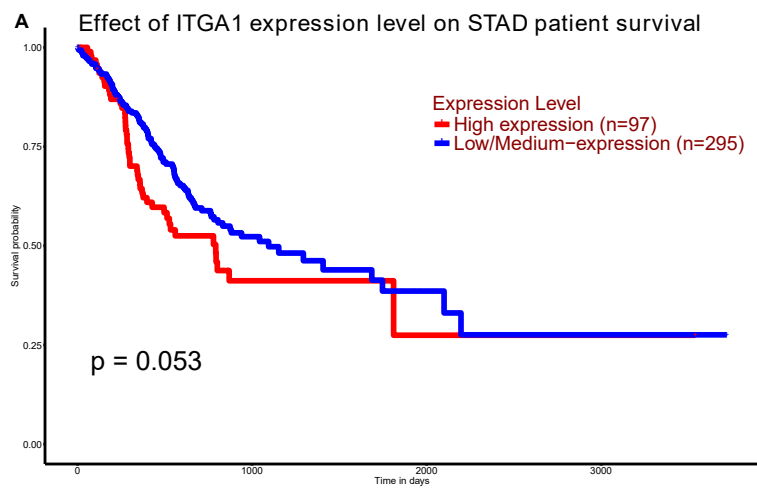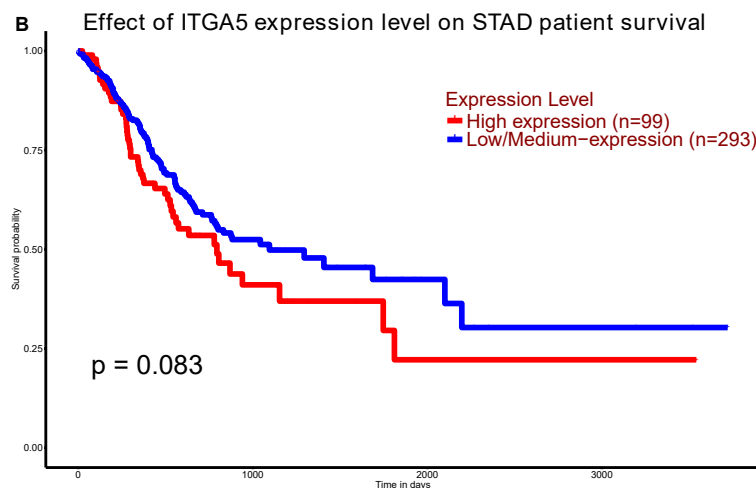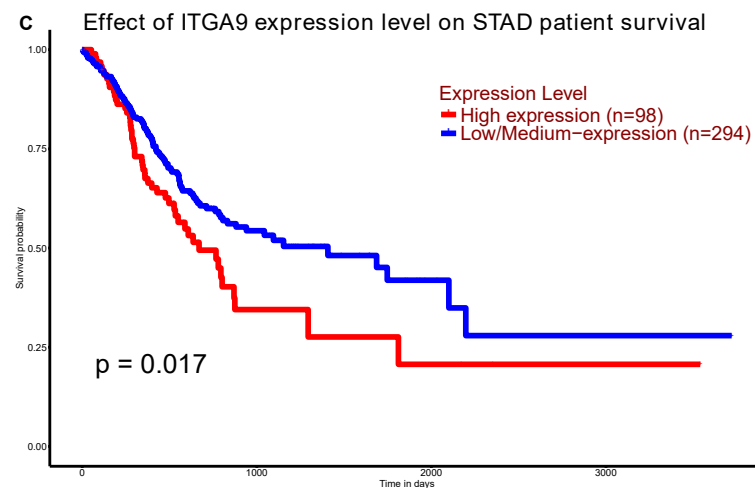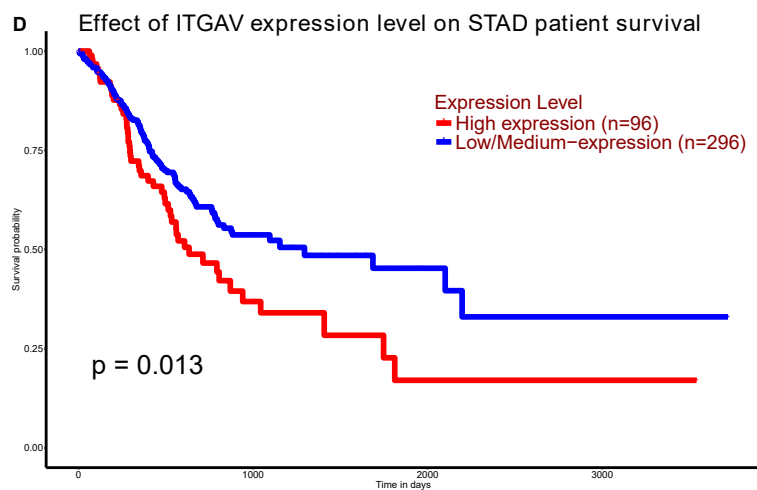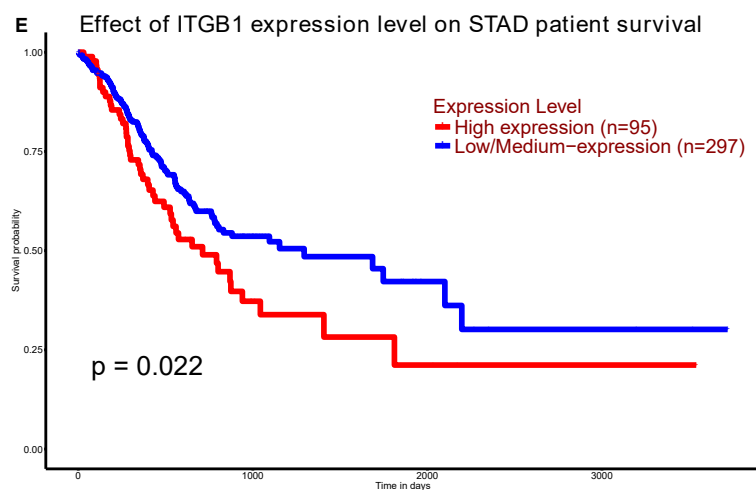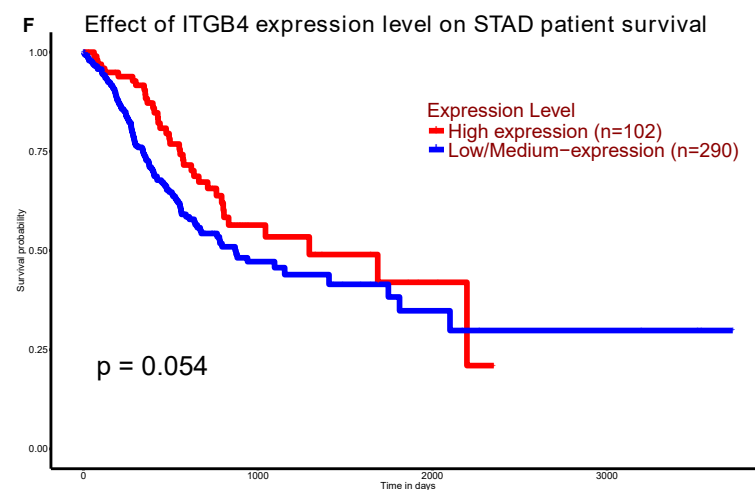

# STs

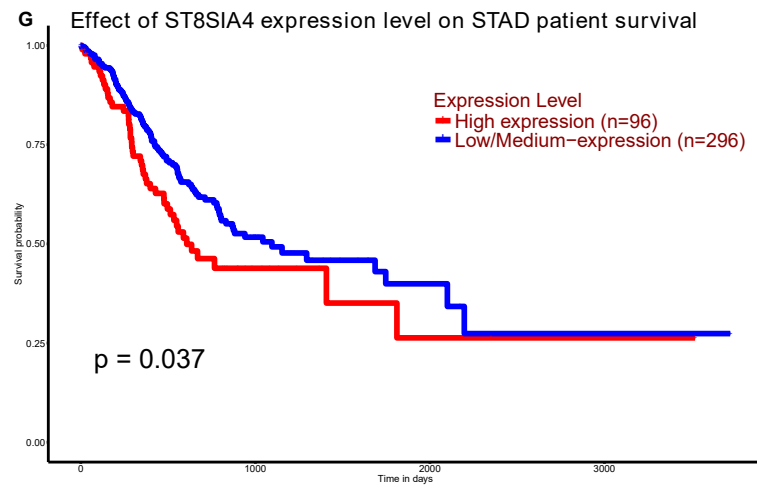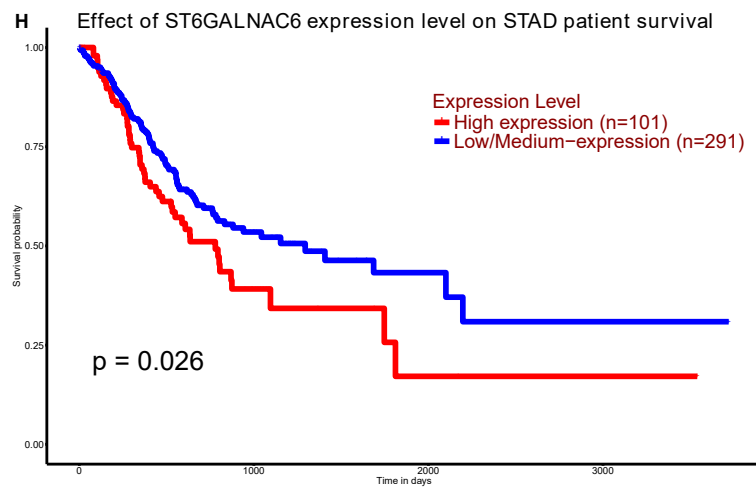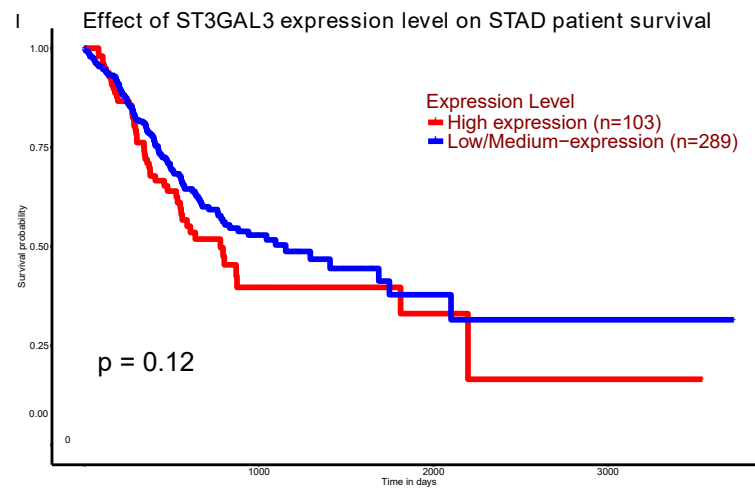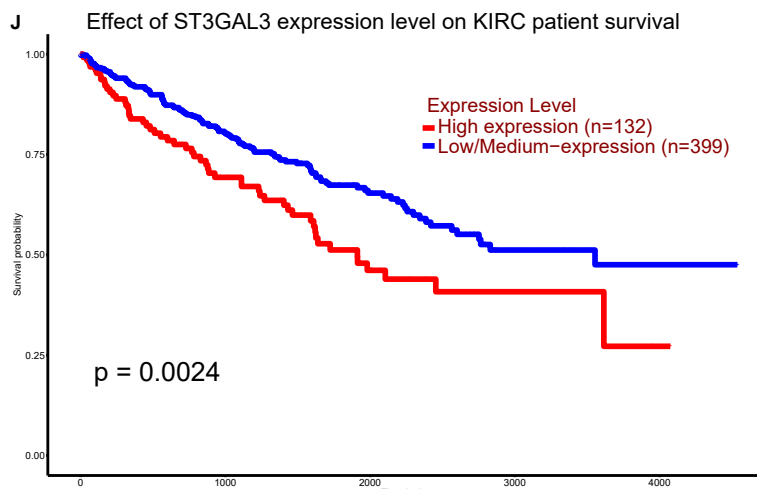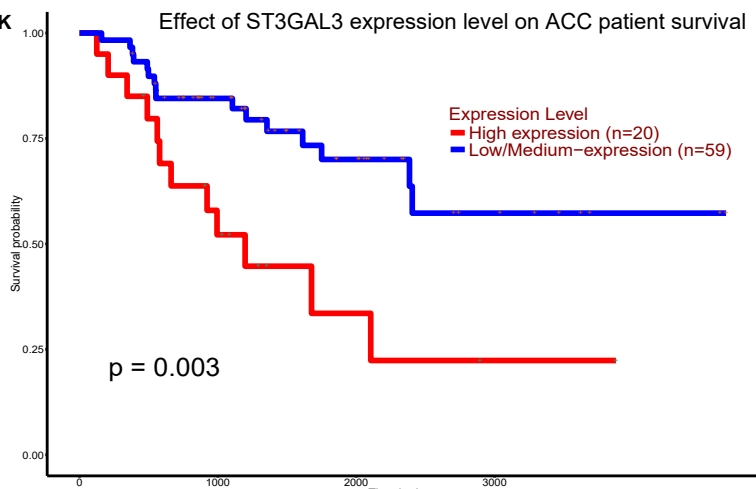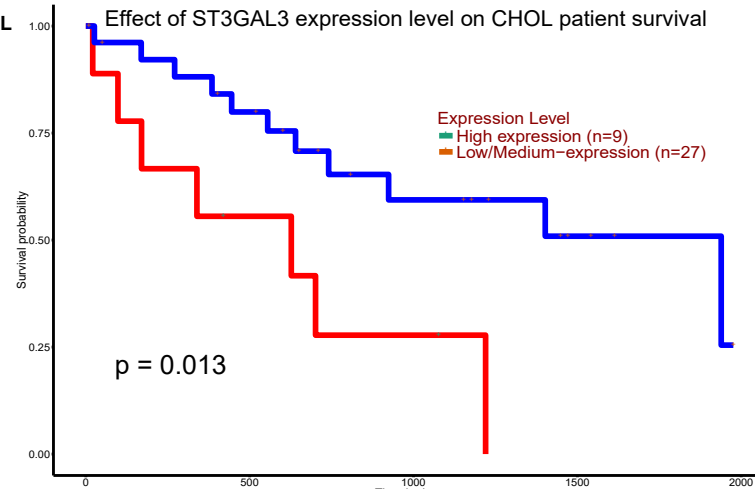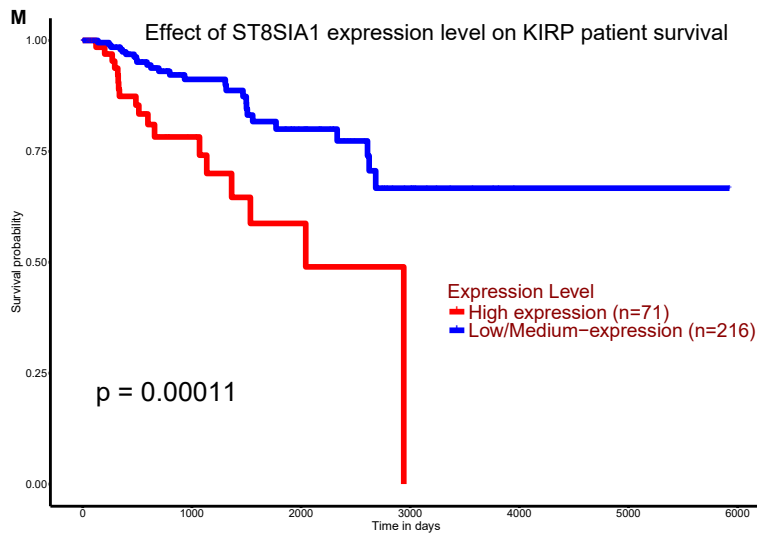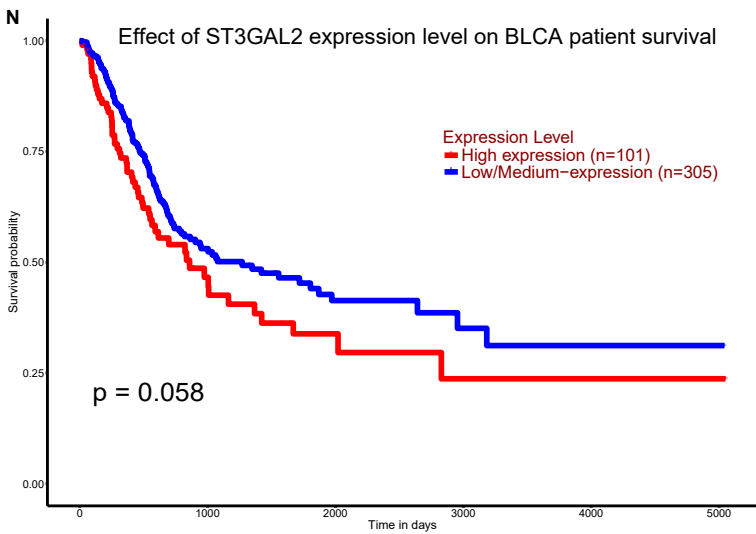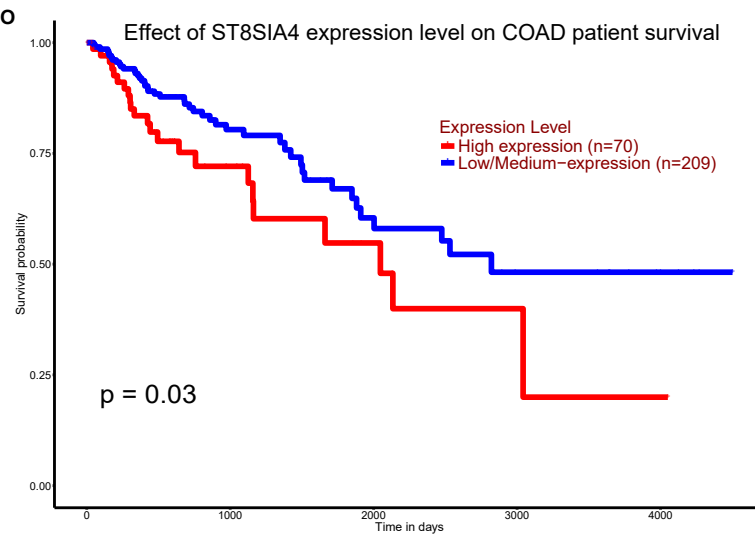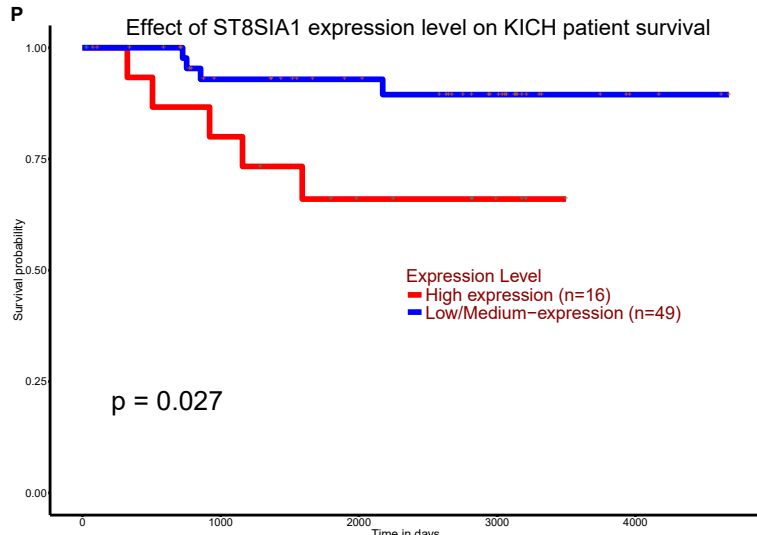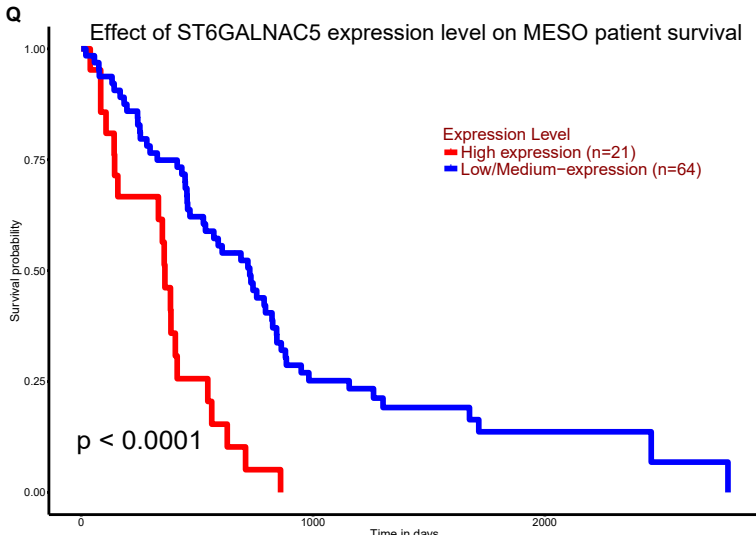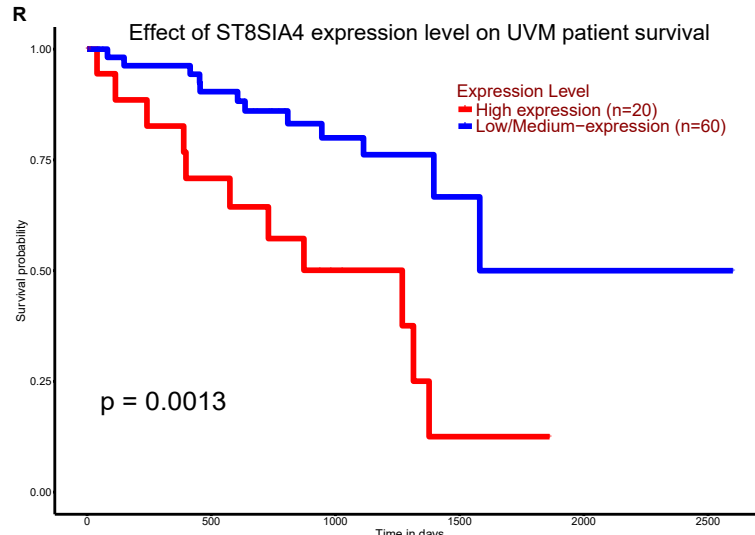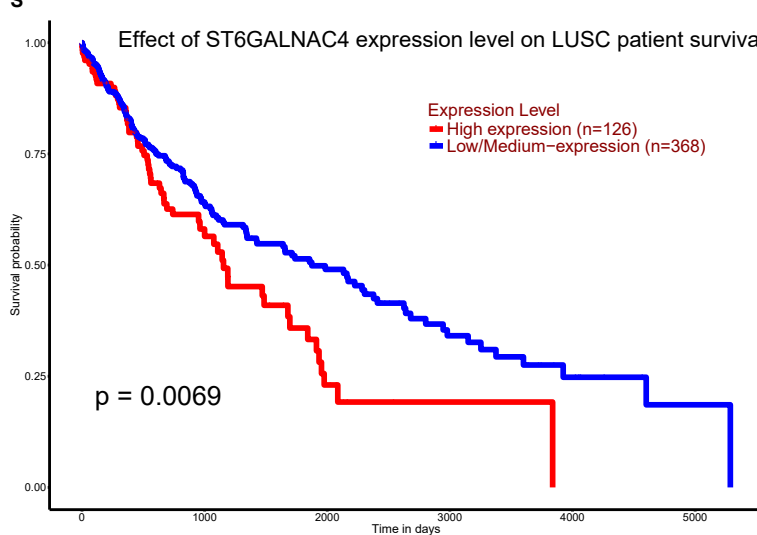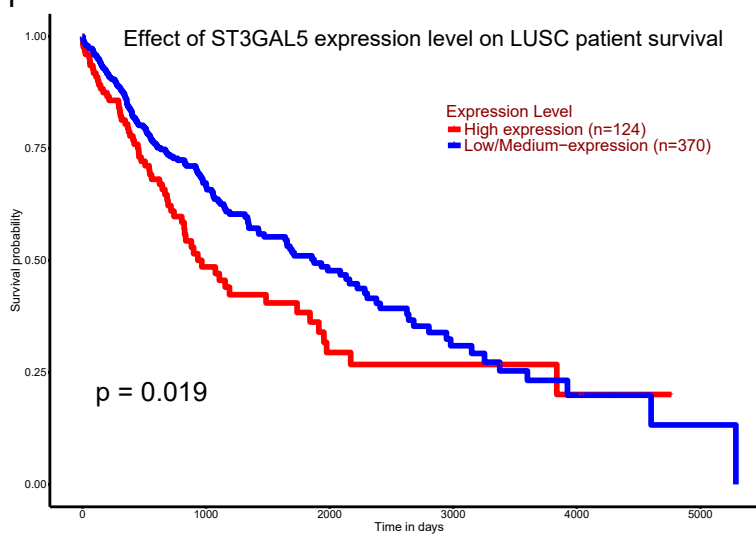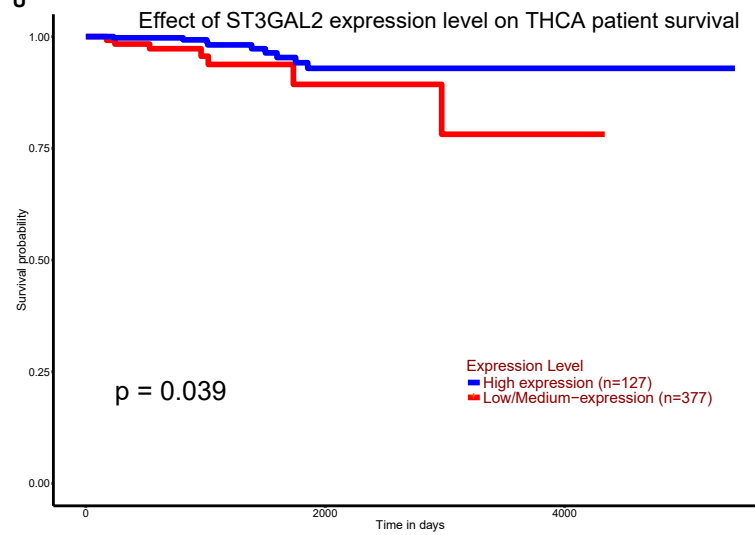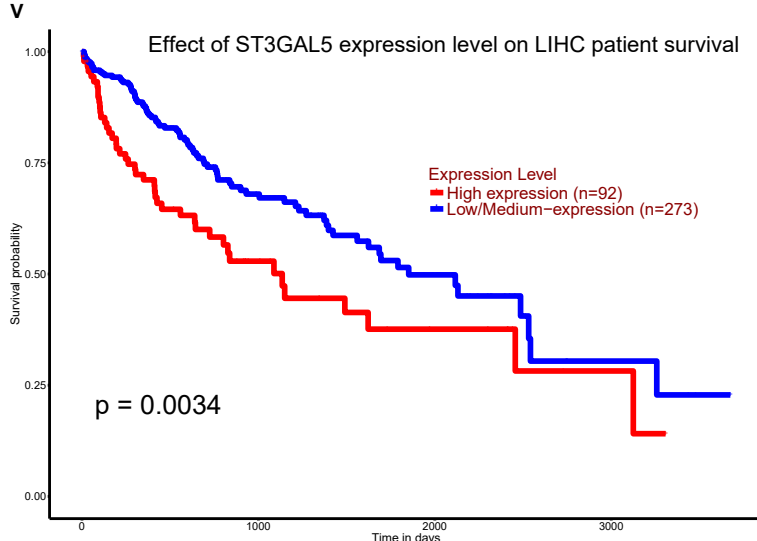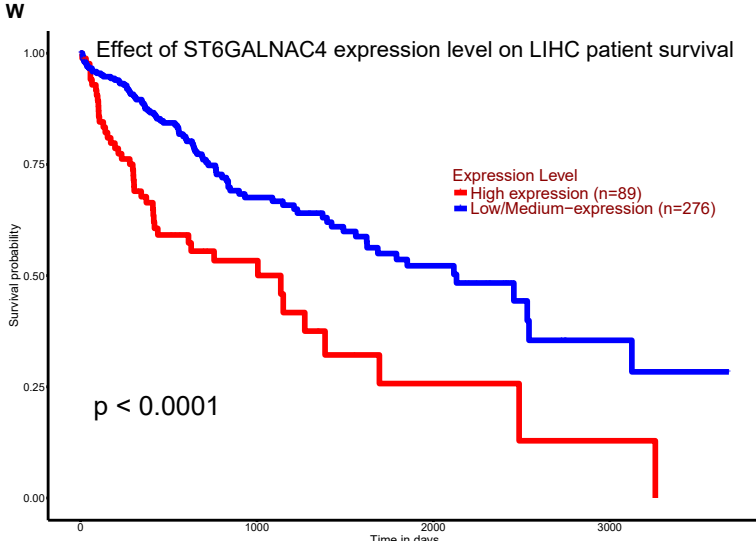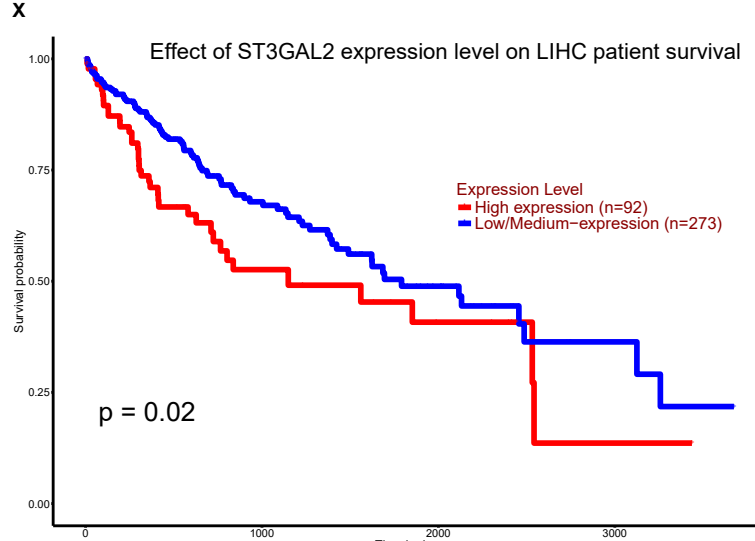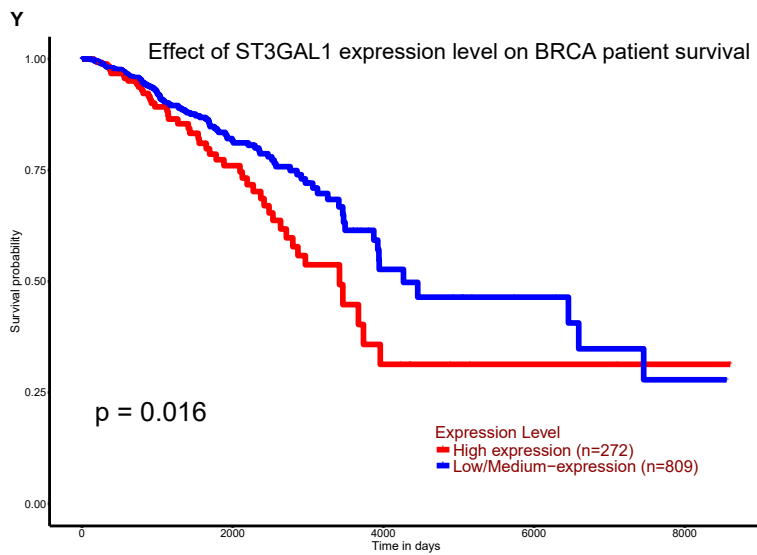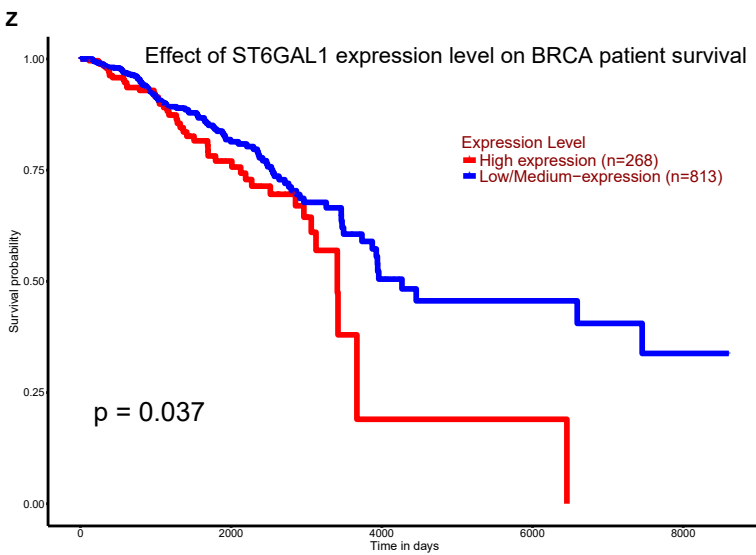

Supplement: nwac177_Supplemental_Files [file nwac177_supplemental_files.zip › Figure_S2-UALCANsurvival(supplementary_data).pdf]

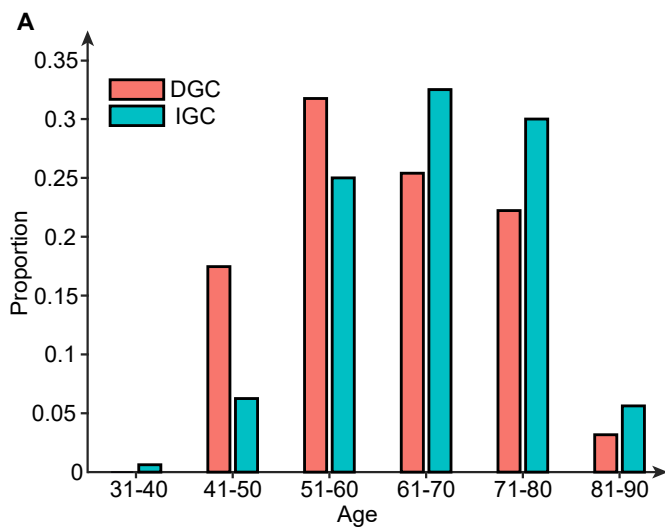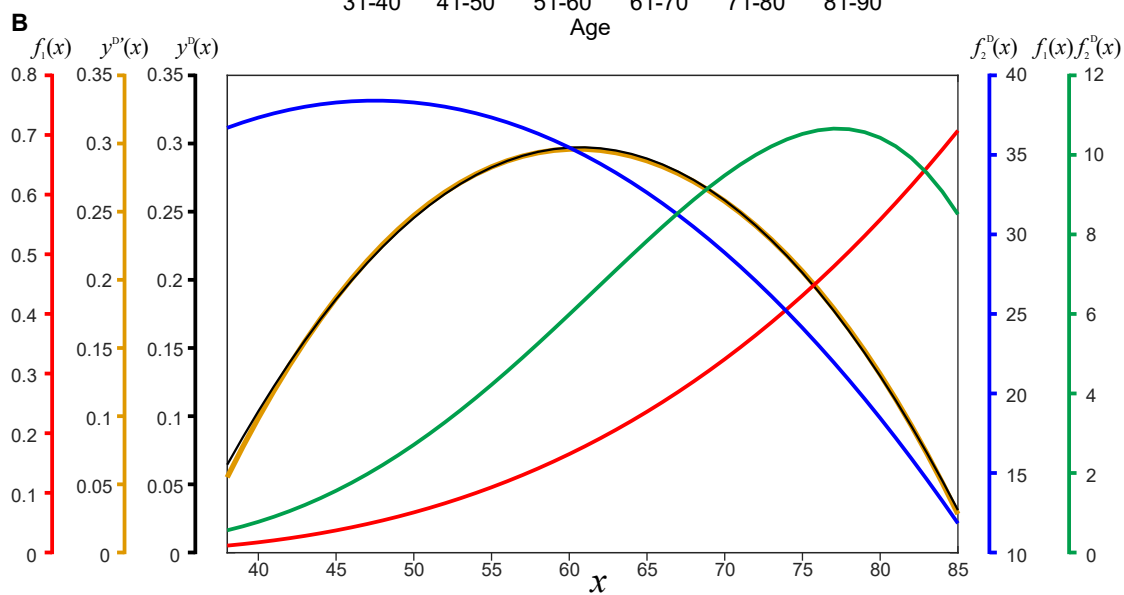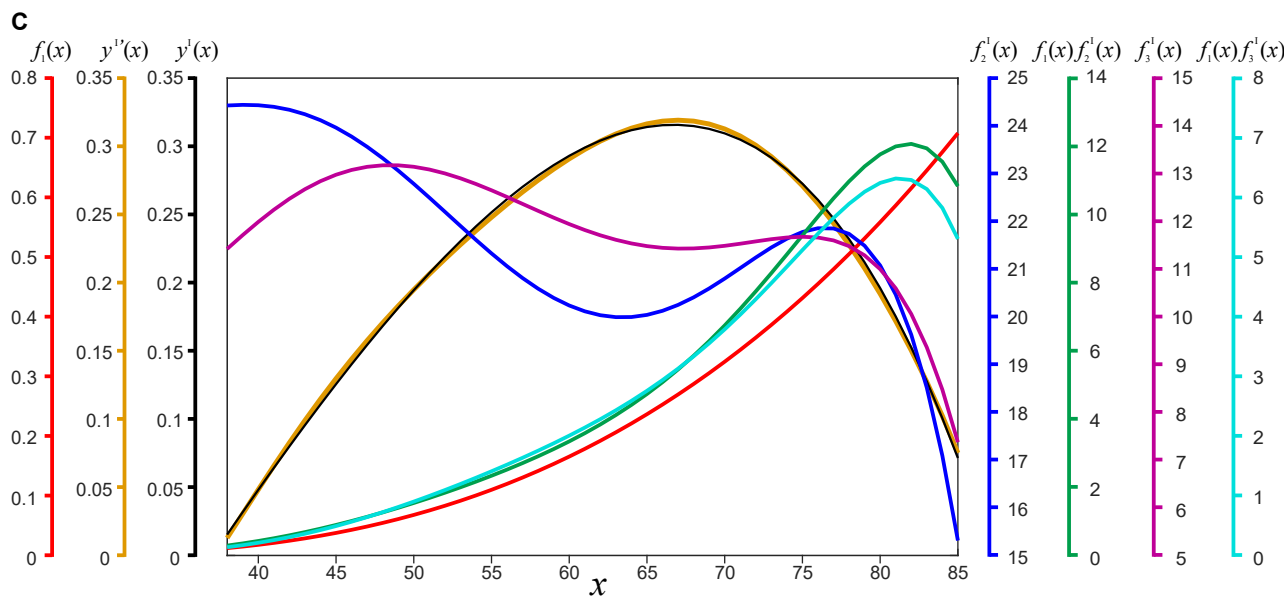

Supplement: nwac177_Supplemental_Files [file nwac177_supplemental_files.zip › Figure_S3-Modelfitting-DGCIGCOccurrenceRates(supplementary_data).pdf]
